# A Novel Mechanism of PPAR Gamma Induction via EGFR Signalling Constitutes Rational for Combination Therapy in Bladder Cancer

**DOI:** 10.1371/journal.pone.0055997

**Published:** 2013-02-08

**Authors:** Jose Joao Mansure, Roland Nassim, Simone Chevalier, Konrad Szymanski, Joice Rocha, Saad Aldousari, Wassim Kassouf

**Affiliations:** McGill Urologic Oncology Research, Division of Urology, McGill University Health Center, Montreal, Quebec, Canada; Wayne State University School of Medicine, United States of America

## Abstract

**Background:**

Two signalling molecules that are attractive for targeted therapy are the epidermal growth factor receptor (EGFR) and the peroxisome proliferator-activated receptor gamma (PPARγ). We investigated possible crosstalk between these 2 pathways, particularly in light of the recent evidence implicating PPARγ for anticancer therapy.

**Principal Findings:**

As evaluated by MTT assays, gefitinib (EGFR inhibitor) and DIM-C (PPARγ agonist) inhibited growth of 9 bladder cancer cell lines in a dose-dependent manner but with variable sensitivity. In addition, combination of gefitinib and DIM-C demonstrated maximal inhibition of cell proliferation compared to each drug alone. These findings were confirmed *in vivo*, where combination therapy maximally inhibited tumor growth in contrast to each treatment alone when compared to control (p<0.04). Induction of PPARγ expression along with nuclear accumulation was observed in response to increasing concentrations of gefitinib via activation of the transcription factor CCAT/enhancer-binding protein-β (CEBP-β). In these cell lines, DIM-C significantly sensitized bladder cancer cell lines that were resistant to EGFR inhibition in a schedule-specific manner.

**Conclusion:**

These results suggest that PPARγ agonist DIM-C can be an excellent alternative to bladder tumors resistant to EGFR inhibition and combination efficacy might be achieved in a schedule-specific manner.

## Introduction

Almost all patients with metastatic bladder cancer succumb to disease, with median survival of 18 months even with the best available chemotherapeutic regimens. As understanding of biology of urothelial carcinoma (UC) improves, novel approaches need to be studied. Two signalling molecules that are attractive for targeted therapy are the epidermal growth factor receptor (EGFR) and the peroxisome proliferator-activated receptor γ (PPARγ). Inhibition of EGFR function is extremely attractive among the wide array of biological targets implicated in urothelial carcinoma (UC) progression. Although mechanism by which EGFR regulates tumor biology in bladder cancer is not clearly defined, it has been demonstrated that EGFR signalling regulates cell survival, proliferation, differentiation, and invasion [1]. Moreover, EGFR is implicated in tumor-induced angiogenesis and metastasis [2]. However, clinical trials with EGFR inhibitors in head and neck, lung, and colon cancer demonstrated that only a minority of patients seemed to benefit from this approach. In context of Non Small Cancer Lung Cells (NSCLC), it was shown that clinical responses were linked to activating mutations within EGFR tyrosine kinase domain, suggesting that better understanding of biological effects of EGFR inhibitors on cancer will help identify tumors that will respond to therapy [3], [4]. Although none of 17 human UC cell lines nor any of 75 primary tumors evaluated at M. D. Anderson Cancer Center contained activating EGFR kinase domain mutations [5], EGFR inhibitors blocked cell cycle progression in 6/17 UC cell lines. We have shown that high EGFR expression is associated with an aggressive phenotype [6] and that modulation of GSK-3β might be a predictor of response to EGFR inhibitors in bladder cancer [7]. We do believe that EGFR remains a strong signalling axis in progression of bladder cancer where its inhibition may benefit selected patients.

Another receptor of interest is PPARγ, a ligand-activated receptor and a member of the nuclear receptor superfamily of transcription factors [8], [9]. Importantly, PPARγ plays an important role in carcinogenesis. PPARγ is highly expressed in tumor samples from different sites, including bladder cancer (reviewed in reference [10]). PPARγ is an interesting target for cancer therapy not only because of its elevated expression in tumors, but also because PPARγ activation results in decreased cell proliferation, decreased G_0_/G_1_ to S phase progression, increased terminal differentiation, and apoptosis [11], [12], [13]. Further, PPARγ agonists are potent angiogenesis inhibitors *in vitro* and *in vivo*, in part due to downregulation of VEGF [14], [15]. Recently, a new class of PPARγ agonists, 1,1-bis(3′-indolyl)-1-(*p*-substitutedphenyl)methanes (PPARγ-active DIM-Cs), has been developed and shown to be significantly more potent than the previous generation of drugs. We published the first report on significant antitumorigenic activity of PPARγ-active DIM-Cs in UC cells *in vitro* and *in vivo* [16]. Use of potent PPARγ-active DIM-Cs was attractive and warrants further evaluation in treatment of UC.

A prior study has shown that PPARγ agonists increase gefitinib’s antitumor activity, possibly mediated through induction of PTEN expression *in vitro* [17]. Additionally, curcumin was shown to induce PPARγ expression and inhibit proliferation in hepatic stellate cells [18]. Others have shown that curcumin can also inhibit EGFR activation and these findings further corroborate the potential crosstalk between the two signaling axes of interest.

The aim of this study is to investigate crosstalk between these two signalling axes as they share some common downstream signalling effectors and evaluate whether combination of PPARγ agonist and an EGFR inhibitor may overcome resistance to EGFR therapy in bladder cancer.

## Materials and Methods

### Cell Culture

The UC cell line 253J B-V was generated from the 253J human UC cell line as previously described [19] and was kindly provided by Dr Colin P.N. Dinney from M.D. Anderson Cancer Center, Houston, Texas. The UM-UC series of urothelial carcinoma cell lines used in this study were genotypically characterised and provided by the Specimen Core of the Genitourinary Specialized Programs of Research Excellence in bladder cancer at M. D. Anderson Cancer Center [20].

### Drugs

Gefitinib (Iressa ZD1839) was supplied by AstraZeneca, London, United Kingdom) and the orally available PPARγ-active DIM-C was generously provided by Dr. S. Safe, Houston, TX as dry powder.

### Cell Proliferation Assay

Bladder cancer cells were treated with different concentrations of gefitinib (0.001 µM to 100 µM) and DIM-C (0.01 µM to 10 µM) in EMEM’s supplemented with 10% FBS for 48 hs Cell proliferation was evaluated using MTT assays (Sigma-Aldrich, Canada). The GI50 value was defined as the mean concentration of drug that generated 50% of growth inhibition.

### Western Blot Analysis

Cells were harvested at ∼75% to 80% confluence in lysis buffer (RIPA) and a cocktail of phosphatase and protease inhibitors (Roche Diagnostics, Germany). Proteins were subjected to SDS-PAGE, transferred onto nitrocellulose membranes (Bio-Rad, Hercules, CA) by semidry electroblotting. Primary monoclonal antibodies [EGF Receptor (15F8), tubulin and b-actin (Cell Signaling Technology, New England, MA)] and PPARγ (sc-7273, Santa Cruz Biotechnology, California, US) were applied to detect bands of interest. Additionally, the following rabbit antibodies from cell signaling were used : Akt; phospho-Akt (Ser473); GSK-3β; phospho-GSK-3β (Ser9); p21 Waf1/Cip1; p44–42 MAPK (Erk1/2); phosphor-p44/42 MAPK (Erk1/2). Anti-rabbit and anti-mouse immunoglobulins (IgGs) coupled to HRP/horseradish were used as secondary antibodies according to the primary antibody.

### Immunofluorescence

Cells cultured in eight-well plastic chambers were washed on ice with cold PBS containing protease inhibitors (Roche Diagnostics, Germany) and fixed with 3.7% paraformaldehyde. Cells were incubated with different concentrations of gefitinib (0, 2, 4 and 8 µM) for 24 hs and then incubated with mouse monoclonal PPARγ primary antibody (1∶50) overnight. Immunofluorescence was revealed using anti-mouse antibodies coupled to FITC (Alexa Fluor 488) or rhodamine (CY3; Invitrogen). 4,6-Diamidino-2-phenylindole (DAPI) was used to stain the nuclei. Photomicrographs were taken with an inverted Olympus IX-81 microscope equipped with a CoolSnap HQ digital camera and the ImagePro+ software (version 5.0.1; Media Cybernetics).

### RNA Isolation and Real Time PCR

Total RNA was extracted from cells using Trizol reagent (Invitrogen, Carlsbad, CA), according to the protocol provided by the manufacturer. The synthesis of cDNA was performed using the Quantitec Reverse Transcription Kit (Qiagen, Mississauga, ON). For RT-PCR amplification, validated primers from Qiagen (Hs_CEBPB_2_SG QuantiTect Primer Assay QT00998494) were used. No genomic DNA contamination or pseudogenes were detected by PCR without the reverse transcription step in the total RNA used. β actin was used as an internal control. The reactions started at 95°C for 10 min, followed by 40 cycles of 95°C for 10 s, 60°C for 20 s. Melting peaks of PCR products were determined by heat-denaturation over a 35°C temperature gradient at 0.2°C/s from 60 to 95°C. The cycle numbers crossing an arbitrary threshold (*C*t) were determined using MyIQ system software, version 1.0.410 (BioRad, CA, U.S.A.). Fold change in target mRNA relative to β actin was calculated as follow: Fold change = 2^−ΔΔ*C*t^ where ΔΔ*C*t = (*C*t _target_ -*C*t β actin)_time *X*_ - (*C*t _target_ - *C*t β actin)_time 0_ Time *X* is time point after 3 hs gefitinib. Time 0 represents the experiment starting time (no drug added).

### Bladder Tumor Xenografts

Female nude mice (purchased from Charles Rivers, Wilmington, MA) were injected subcutaneously with the KU-7 cells (10^6^ cells per injection). Animals of each series (10 mice per group) were randomised and assigned to treatment and a placebo arms. DIM-C was given 60 mg/kg 3 times per week and gefitinib was given 2 mg/day, 5 times per week. All drugs and placebo were given by oral gavage. Treatment was continued for 4 weeks and subsequently tumors were harvested and weighted. Tumors were snap-frozen in liquid nitrogen for further analysis.

This study was carried out following the Standard Operating Procedures for Care and Use of Laboratory Animals of the McGill University Animal Care Committee. The protocol was approved by the Facility Animal Care Committee of the Research McGill University Health Center (Permit Number: 5428). All surgery was performed under sodium pentobarbital anesthesia, and all efforts were made to minimize suffering.

### Immunohistochemistry

Serial sections of tumor xenografts from mice treated with placebo and combination treatment (gefitinib plus DIM-C) were incubated overnight at 4°C, with primary specific antibodies against PPARγ (sc-7273 mouse monoclonal IgG_1_ antibody 1∶1000 dilution, Santa Cruz, CA, USA), p21 (12D1 rabbit antibody 1∶100 dilution, cell signaling, MA, USA). Goat polyclonal anti-rabbit IgG secondary antibody, conjugated with HRP was added and incubated for 1 h at room temperature. Color development was performed with DAB substrate (Sigma Aldrich, Canada), according to manufacturer’s instructions. Immunostaining was evaluated in a semiquantitative method based on the average of five foci on percentage of viable cells showing positive expression. Specimens were scored based on the intensity of antibody nuclear and cytoplasmic staining in each slide. Values were compared using unpaired Student’s t test.

### Microarray Analysis

Bladder tumors xenografts, were sectored stained by hematoxilin and eosin and the tumors were mapped for further isolation. Total RNA was extracted as previously described. RNA was quantified using a NanoDrop-ND1000 spectrophotometer (Thermo Fisher Scientific, Wilmington, DE) and quality was monitored with the Agilent 2100 Bioanalyzer (Agilent Technologies, Genome Quebec Innovation Center, CA ). Microarray analyses were performed at McGill University and Genome Quebec Innovation Center, using Illumina BeadArray™ technology. The HumanHT-12 Expression BeadChip™ was used and contained more than 22,000 probes from the NCBI RefSeq database, which provides higher throughput processing of 12 samples per chip. There is a coverage of >99.99% of all bead types on any given HumanHT-12. TotalPrep RNA Amplification kit from Ambion was used to perform one round of amplification from 50–500 ng of total RNA. The cDNA synthesis and *in vitro* transcription amplification were followed by hybridization. The BeadChips were imaged using Illumina's BeadArray or iScan reader. Statistical analysis and visualization of data from microarray experiments was performed using the software package FlexArray version 1.6 developed and provided by Genome Quebec. Functional and signalling pathway analyses were assessed using Ingenuity Pathway Analysis (IPA) software.

### Statistical Analysis

All data were analyzed using the STATA version 10.0 software. Results from *in vivo* were compared using repeated measure ANOVA and Fischer’s exact test. P<0.05 was considered to be statistically significant.

## Results

### Baseline Expression of PPARγ and EGFR in a Panel of Urothelial Carcinoma Cell Lines

**Figure 1:**
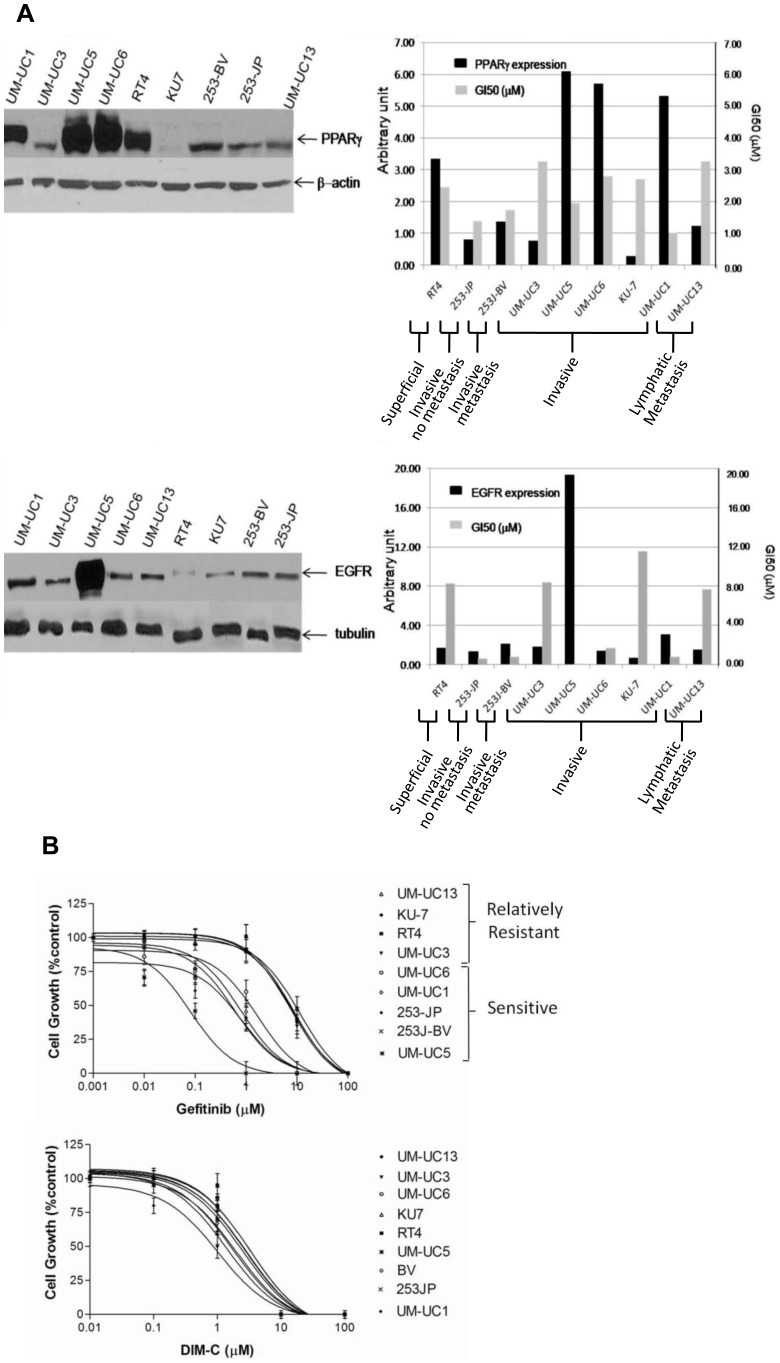
Baseline expression of PPARγ and EGFR. (A) Expression of PPARγ and EGFR relative to endogenous levels of β-actin and tubulin, respectively, and represented in units among 9 bladder cancer cell lines reflecting different stages of the disease (from Superficial to Invasive & no Metastasis, Invasive and Lymphatic Metastasis). (B) Dose-response of bladder cancer cell lines to PPARγ agonist (DIM-C) and EGFR inhibitor (gefitinib). The GI50 value was defined as the mean concentration of drug that generates 50% of growth inhibition as compared to controls.

We have previously reported that inhibition of EGFR signalling axis and activation of PPARγ axis are both effective in significantly inhibiting proliferation of human carcinoma cells through different pathways, in part converging to PI3K/Akt, cyclin D1, and cyclin-dependent kinase inhibitors [7], [16]. In our previous work, we have shown significant expression of the HER family members across various UC cell lines [7]. To further investigate for interaction between the two signalling axes, we first screened to characterize the levels of EGFR and PPARγ expression across a panel of 9 UC cell lines. As revealed in Figure 1 A, all the cell lines tested expressed various levels of EGFR and PPARγ. We did not demonstrate a correlation between baseline levels of expression and stage of disease of which the 9 cell lines were derived from (from superficial to invasive to metastatic). We have also determined the dose response of among the urothelial carcinoma cell lines (UM-UC1, UM-UC3, UM-UC5, UM-UC6, UM-UC13, RT4, 253JP, 253J-BV, KU7) to different concentrations of EGFR inhibitor (gefitinib) and PPARγ agonist (DIM-C) after 72 hs of treatment (Figure 1 B). We were able to stratify several UC cell lines ranging from highly sensitive to relatively resistant to EGFR inhibition, while no significant changes were observed to justify a stratification in response to DIM-C. Of note, UC5, the most sensitive cell line to gefitinib, is different from the rest of the cell lines as it contains EGFR gene amplification.

### Effects of Combined EGFR Inhibitors and PPARγ Agonists Therapy

**Figure 2:**
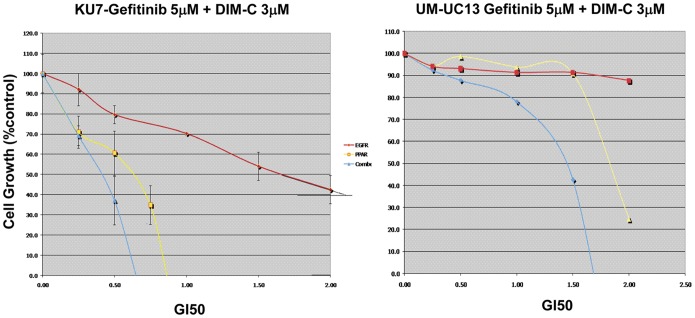
Antiproliferative effects of combined therapy. Growth was monitored by MTT assays. Cells were treated with gefitinib 5 µM and DIM-C 3 µM and compared to each drug alone. Red: gefitinib; Yellow: DIM-C; Blue: gefitinib+DIM-C.

We investigated the antiproliferative effects of combined therapy, gefitinib and DIM-C, compared to each drug alone on bladder cancer cell growth *in vitro*. Growth and cell proliferation were monitored by MTT assays and conducted on two relatively EGFR-resistant cell lines (KU7 and UM-UC13). Cells were treated for 72 hs and used a fixed-ratio of different fractions of GI50 (0.25, 0.5, 1.0 and 2.0) of each drug alone according to median effect method. Maximal inhibition of cell proliferation was demonstrated in the combined treatment compared to either drug alone (Figure 2). DIM-C rendered the resistant cells sensitive to EGFR inhibition.

### Effect of Combination on the EGFR Downstream Signaling and PTEN Expression

**Figure 3:**
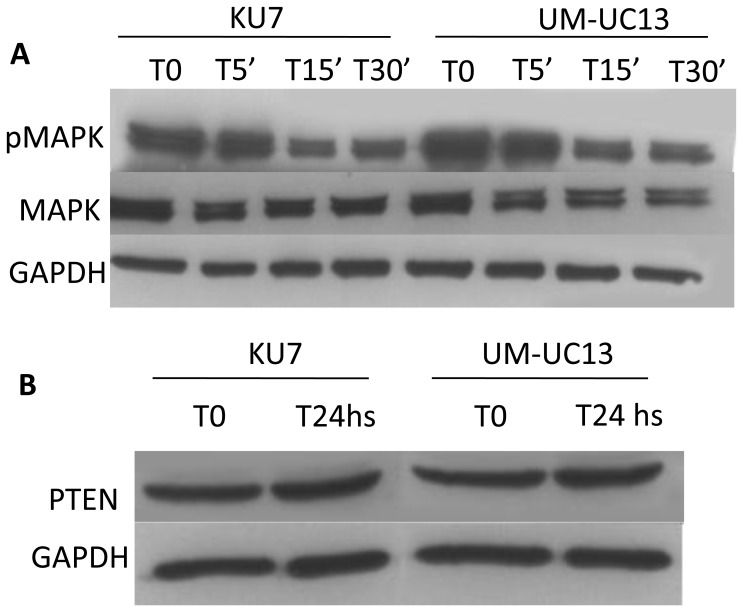
Effect of combination therapy on EGFR downstream signaling. (A) Phosphorylation pattern of p42/44 MAPK (Erk1/2) in cells treated with gefitinib 5 µM and DIM-C 3 µM for 5, 15 and 30 minutes. T0 is the untreated control. Whole-cell lysates were immunoblotted with phosho-p42/44 MAPK and p42/44 MAP. GAPDH was used as loading control on the Western blotting. (B) Comparison of PTEN expression in cell lines treated with gefitinib 5 µM and DIM-C 3 µM for 24 hs. T0 is the untreated control.

Binding of EGFR to its ligand leads to activation of various signals, including the Ras/Raf/mitogen-activated protein kinase pathway (MAPK) [21]. Therefore, we investigated the effect of combination in the phosphorylation status of p42/44 MAPK (Erk1/2). As seen in Figure 3A, a significant time course deactivation of the Erk pathway was observed, compared to control, among cells treated with gefitinib 5 µM and DIM-C 3 µM. Additionally, as previously mentioned, expression of PTEN increases in non-small cell lung cancer (NSCLC) treated with the PPARγ agonists, rosiglitazone [17]. Therefore, we also investigated the effect of combination treatment in PTEN expression. As shown in Figure 3B, no difference in PTEN expression was observed, as compared to control, when cells were treated with combined gefitinib and DIM-C for 24 hs.

### Effects of Treatment on Bladder Tumor Growth in vivo

**Figure 4:**
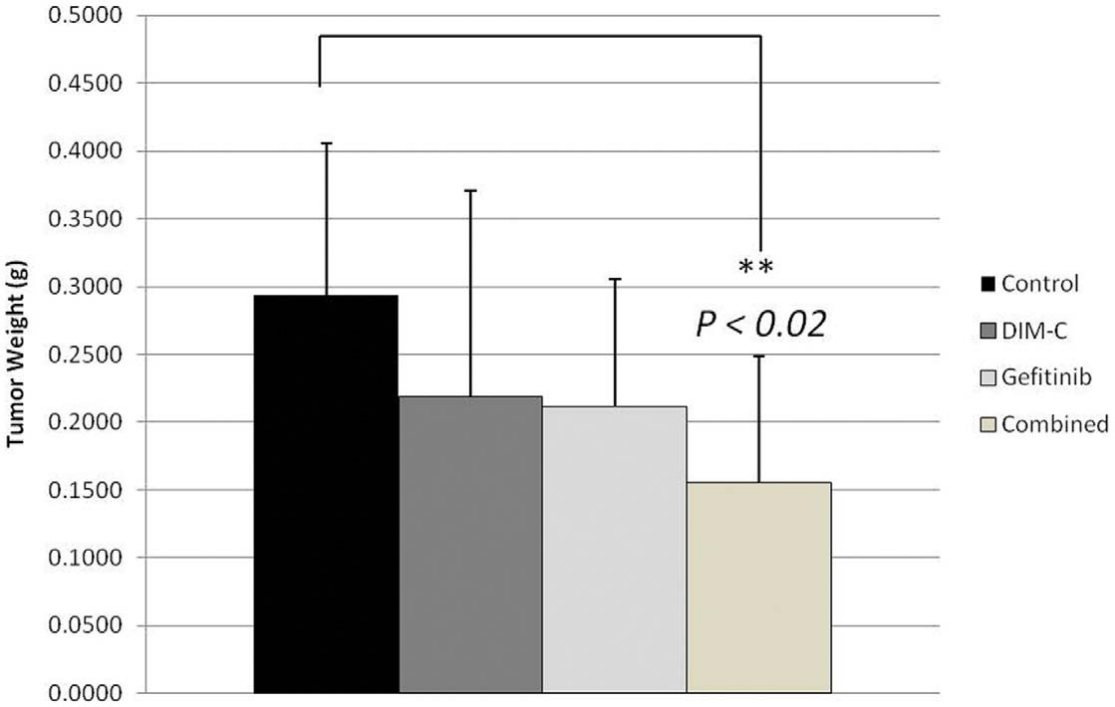
Effects of combination therapy *in vivo*. Bladder tumor growth of combination treatment arm compared to control arm (P<0.02). Ten mice per group were treated with placebo; DIM-C was given 60 mg/Kg 3 times per week; Gefitinib was given 2 mg/day, 5 times per week. All drugs were administrated by oral gavage. Treatment was continued for 4 weeks.

**Figure 5:**
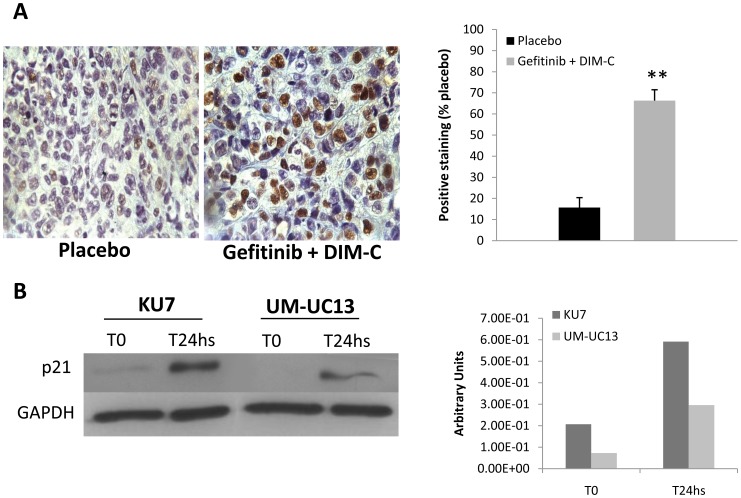
Effects of combination therapy on p21 expression *in vivo* and *in vitro*. (A) Immunohistochemistry (IHC) staining for p21 in tumor xenograft tissues. Mice were treated with placebo or combination therapy (Gefitinib 2 mg/day, 5 times per week and DIM-C 60 mg/Kg, 3 times per week). Graphic on the right side represents quantification of positive staining cells. (B) In vitro expression of p21 in Western blot of lysate cells treated with gefitinib 5 µM and DIM-C 3 µM for 24 hs. Graphic on the right side, represents quantification of p21 expression related to GAPDH.

To evaluate whether these findings can be translated *in vivo*, nude mice were inoculated subcutaneously with relatively resistant bladder cancer cells (KU-7 cells). The mice were treated with targeted agents via oral gavage (PPARγ-active DIM-Cs given 60 mg/kg 3 times per week, gefitinib given 2 mg/day 5 times per week, or both) for 4 weeks. As shown in Figure 4, tumor weights were markedly reduced in combined group in contrast to each drug alone when compared to control (*p*<0.02). These findings suggest combined treatment has a better antitumor activity. Furthermore, gene expression profiling analysis of the bladder tumor xenografts, showed that several genes involved in cell cycle, cell death, cellular growth and proliferation were differently expressed in the combined treatment group as compared to the control group. (Table 1). Remarkably, cyclin-dependent Kinase inhibitor (CDKN1A or p21), which functions as a regulator of cell cycle progression at G1, was significantly upregulated (Fold change 2.6, *p* value <0.02) and this was validated by immunohistochemistry showing higher percentage of p21 positive cells in the combined arm *in vivo* (66% vs 15%, p<0.001) (Figure 5A) as well as *in vitro* (Figure 5B). Interestingly, it has been reported that PPARγ plays an important role mediating the differentiation-dependent cascade expression of cyclin-dependent kinase inhibitors, thereby providing a molecular mechanism coupling growth arrest and adipocyte differentiation [22].

**Table 1:** Analysis of molecular pathways and functions of the differentially expressed mRNAs of combined treatment compared to control group in bladder tumor xenografts.

Molecules in Network	*P* - Value	Top Functions
AXIN2, BACH2 **CDKN1A**, DUSP6,DYRK1B, FOXO4, HAS2, MED16, MMP9, NCOR2, P38 MAPK, PDGF BB,PLTP, SCGB1A1, STK39, SYNE1,TNFSF11, VTN, XAF1	10E-38	Cell Cycle, Tumor Morphology,Cell Morphology
ALDH3A1, AMT, APOF, BDP1,CCDC11, CDK18, **CDKN1A**, Cyclin D1/cdk4, NEURL2, NKAP, POMT1,STX17, TAPBPL, VEZT	10E-23	Cell Death, Genetic Disorder,
ARMCX3, CAMTA2, CDC42EP1,FKBP2, HNRNPM, HSPBP1, HTR1EKCNAB1, PIP4K2B, SRGAP2, TUSC3,WDR17, ZFP91	10E-23	Cell Cycle, Tumor Morphology,Cellular Assembly andOrganization
DLX2, DLX3, EXOC1, FANCD2,HAS2, HAUS6, HEATR3, IL1RAPL1,MDN1, MYC, OSBPL1A, PROCKLE1,PXMP4	10E-22	Gene Expression, Cancer and Immunological Disease
ARPC3, CFB, CFP, COL12A1, DDAH2,KCNK6, KCTD7, KLHL4, PILRA,PTPRB, SLC14A1, SMPDL3B	10E-17	Cell Signaling, and Inflammatory Response
MYCN	10E-2	Cancer, Cell Cycle, Cell-To-CellSignaling and Interaction

IPA analysis was performed in order to identify the molecular pathways and functions of the differentially expressed mRNAs of combined treatment compared to control group in bladder tumor xenografts. Most significantly enriched groups relating to molecular and cellular functions are shown. The networks were generated on the basis of the published literature and ranked by the P-value calculated by Fisher’s exact Test.

### EGFR Inhibition Induces Gene Expression of PPARγ in a Dose-dependent Manner

**Figure 6:**
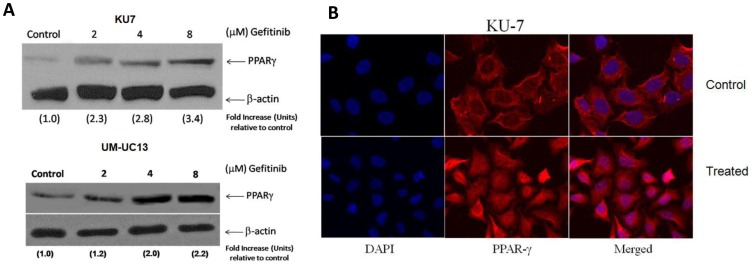
Induction of PPARγ expression in response to different concentrations of gefitinib. (A) Fold increase relative to control was determined after normalization with β-actin as external loading control. (B) Upregulation and nuclear accumulation of PPARγ following treatment with gefitinib (24 hs).

**Figure 7:**
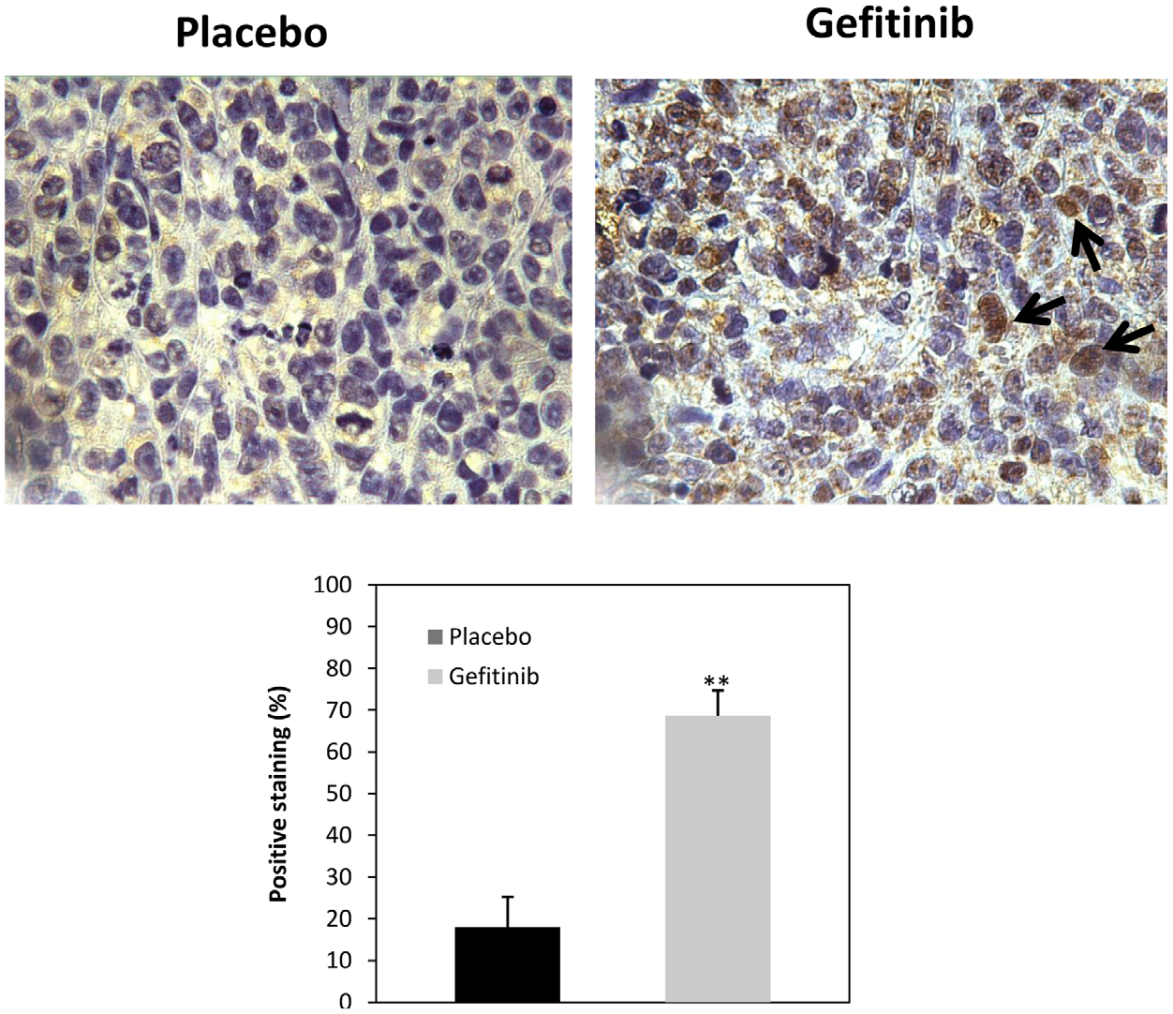
Immunohistochemistry (IHC) staining for PPARγ in tumor xenograft tissues Mice were treated with placebo or gefitinib (Gefitinib 2 mg/day, 5 times per week). Graphic on the lower level represents quantification of positive staining cells. Black arrows indicate nuclear staining.

We evaluated the effect of gefitinib on members of the PPARγ signalling axes. Two resistant cells (KU-7 and UM-UC13) were treated with different concentrations of EGFR inhibitor for 24 hs. PPARγ expression was determined by western blotting analyses as previously described. As shown in Figure 6A, a dramatic induction of PPARγ expression was observed in both cell lines, in a dose dependent manner, followed EGFR inhibition. Interestingly, a nuclear accumulation of PPARγ was also observed following its upregulation, which is in fact the active form of the receptor. (Figure 6B). These findings were confirmed *in vivo*, as seen in Figure 7, that shows PPARγ expression is higher among the xenograft tumors treated with gefitinib as compared to the placebo group. Of note, a nuclear staining was also observed, suggesting a nuclear translocation of PPARγ following induction of its expression.

### Schedule-specific Efficacy of Combination Therapy

**Figure 8:**
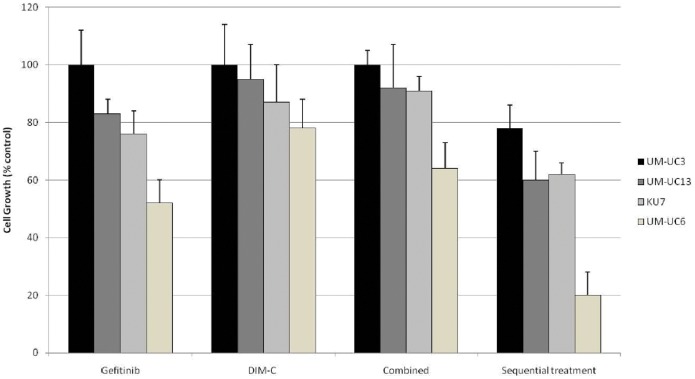
Schedule-specific efficacy of combination therapy. Three relatively resistant cell lines to gefitinib (UM-UC3, UM-UC13, and KU-7) and one sensitive (UM-UC6). Growth (MTT assays) after 48 hs treatment. Gefitinib: 2 µM. DIM-C: 2 µM.

If cells are sensitized to EGFR inhibition via induction of PPARγ expression, then one would expect that efficacy of combination therapy may also be significantly affected and improved by sequence of administration of gefitinib and DIM-C. In fact, we observed marked effect on proliferation among three relatively resistant and one sensitive cell lines to gefitinib (KU-7, UM-UC3; UM-UM13) when cells were pre-treated with gefitinib for 24 hs to allow for induction of PPARγ expression compared to when the cells were simultaneous exposure to gefitinib and DIM-C (Figure 8). These findings strongly suggest PPARγ agonist could significantly sensitize UC cell lines, particularly those that were resistant to EGFR inhibition, in a schedule-specific manner and provide an excellent potential for combination therapy.

### C/EBPβ Expression After Gefitinib Induced-PPARγ Expression

**Figure 9:**
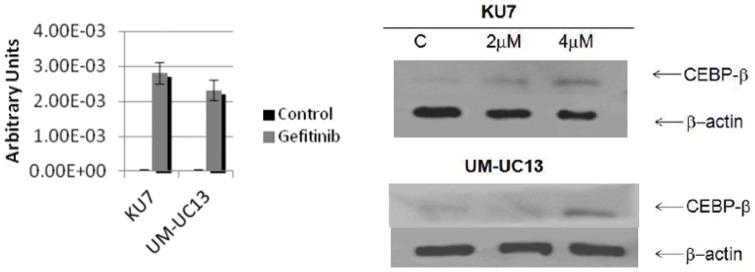
mRNA expression of CEBPβ after treatment with gefitinib. (A) RT-PCR of cells treated with gefitinib (B) Western blot of CEBPβ expression in KU-7 and UM-UC-13 cell lines in response to different concentrations of gefitinib.

Considerable evidence indicates that CCAAT/enhancer-binding protein beta (C/EBPβ) acts as a transcriptional activator for PPARγ genes [23], [24]. This belief is supported by the fact that the proximal of its promoters possess C/EBP regulatory elements that are essential for transactivation of PPARγ promoter-reporter transgenes. Here we report compelling evidence that sequential induction of PPARγ expression by EGFR inhibition is mediated by C/EBPβ (Figure 9). When cells were pre-treated with gefitinib at the same concentrations used to induce expression of PPARγ, an increase in C/EBPβ was also observed suggesting gefitinib induced-PPARγ expression may be mediated by C/EBPβ.

## Discussion

Results from a large body of preclinical studies and clinical trials suggest that targeting EGFR represents a significant contribution to cancer therapy. However, the issue of constitutive resistance in a large number of patients and the development of acquired resistance in the responders remains an unexplored subject of investigation. Cancer cell resistance to EGFR antagonists could be due to several reasons, such as genetic alterations, which enable them to have an intrinsic resistance to anticancer drugs. In addition, several different molecular changes important in EGFR dependent or -independent cellular signalling pathways could be responsible for the development of resistance to these inhibitors. For instance, we have previously shown uncoupling of EGFR with mitogenic pathways can cause resistance to EGFR antagonists [7]. Currently, combined therapy has become a breakthrough in treating cancer. In a range of tumor entities, such approach has produced impressive results. Combination therapy of PPARγ agonists and other agents has been shown to be more effective than using either agent alone. [25], [26]. In bladder cancer cells *in vitro* and in bladder tumor *in vivo*, we demonstrated that PPARγ active DIM-Cs showed significant anti-tumorigenic activity and were more potent inhibitors of bladder cancer growth when compared with rosiglitazone, the currently used synthetic PPARγ agonist [16]. Taken together, combined targeting of both EGFR and PPARγ axes can reveal promising molecules to target in bladder cancer. However, our results have shown that human urothelial cancer cell lines display marked heterogeneity towards sensitivity to EGFR inhibitors. Of high interest, the levels of expression of EGFR and PPARγ varied significantly among the different cell lines but did not correlate with stage of disease (range from superficial papillary to invasive to metastatic tumors). Furthermore, this correlation was not perfect as well to sensitivity either to EGFR inhibitor or PPARγ agonist with exception of UM-UC5 that shows high levels of EGFR expression and display high sensitivity to EGFR inhibitor. These findings corroborate results from other groups that have reported, in a panel of 17 human bladder cancer cell lines, that despite the strong correlation among gefitinib-responsiveness, EGFR surface expression and p27Kip1 protein expression in the most responsive lines, gefitinib-responsiveness was not as tightly linked to surface EGFR expression within the panel of cell lines as a whole [27]. These results are remarkable but remain unclear whether baseline expression could predict EGFR dependent growth in bladder cancer. Conversely, when two resistant cell lines (KU-7 and UM-UC13) were treated with fixed-ratio of different fractions of GI50 of each drug alone (gefitinib or DIM-C), combination therapy potentially exerts additive inhibition of UC cell proliferation. These results provided further insight into the potential of combined therapy to overcome resistance to either drug alone, particularly to EGFR inhibitor. Indeed, our *in vivo* findings, showed positively the effects of combined EGFR inhibitors and PPARγ agonists on the growth of human urothelial tumors. In fact, xenografts nude mice with the relatively resistant bladder cancer cells (KU-7 cells) showed a markedly reduced tumor weight in combined group, in contrast to each treatment alone when compared to control. These findings indicate that even relatively resistant cells, *in vitro*, demonstrated sensitivity *in vivo* in the combined treatment, suggesting PPARγ agonist could potentially be used to sensitize bladder cancer cell lines that were resistant to EGFR inhibition.

Recently, curcumin was shown to induce PPARγ expression in hepatic stellate cells and inhibit cell proliferation potentially *via* inhibiting EGFR activation [28]. In this study, Zhou et al, reported that interruption of the PDGF and EGF signalling pathways by curcumin, stimulates gene expression of PPARγ in activated Hepatic Stellate Cells (HSC), leading to the reduction in cell growth, including induction of cell arrest and apoptosis. Similarly, we observed an induction of PPARγ expression upon inhibition of EGFR in resistant KU-7 and UM-UC13 cells**.** This was an attractive result, since induction of PPARγ expression was observed in a dose dependent manner, and was followed by a nuclear accumulation of PPARγ, which is, in fact, the functional form of the receptor. Our novel observation reflects that efficacy of combination therapy may also be significantly affected and improved by sequenced administration of gefitinib and PPARγ agonist. In reality, PPARγ agonist markedly sensitized bladder cancer cell lines, particularly those that were resistant to EGFR inhibition, in a schedule-specific manner, suggesting it can be an excellent alternative when cells are resistant to the aforementioned monotherapies. The mechanism of induction of PPARγ gene expression by gefitinib is still not clear. During adipogenesis, considerable evidences indicate that CEBP/β act as a transcriptional activator of PPARγ genes [23]. This belief is supported by the fact that proximal promoters of PPARγ posses C/EBP regulatory elements essential for its transactivation. Moreover, it has been shown that CEBP/β is expressed at early stage subsequently to treatment with differentiation inducers [29] followed by expression of PPARγ. Indeed, our findings shows EGFR inhibition induced CEBP/β expression and interestingly, its upregulation was also observed at early stage after gefitinib treatment, a process very similar to activation of adipogenic genes during differentiation, and which precedes induction of PPARγ gene expression. However, future studies are needed in order to determine the direct role of C/EBPβ in the induction of PPARγ expression mediated by gefitinib. When interpreting our results, it is important to recognize the limitations of preclinical studies. Additionally, long-term use of PPARγ agonist such as pioglitazone has been associated with risk of bladder cancer while short-term use has shown no association [30], [31]. Therefore, despite our promising findings show PPARγ agonists are more effective in combination therapy and particularly render bladder tumor sensitive to EGFR inhibition, a better understanding of the mechanism of activated PPARγ and EGFR inhibition is needed to evaluate the benefits from such therapy in future clinical applications.

## References

[1] Ciardiello F (2000) Epidermal growth factor receptor tyrosine kinase inhibitors as anticancer agents. Drugs 60 Suppl 1: 25–32; discussion 41–22. 10.2165/00003495-200060001-00003 11129169

[2] Mendelsohn J, Dinney CP (2001) The Willet F. Whitmore, Jr., Lectureship: blockade of epidermal growth factor receptors as anticancer therapy. J Urol 165: 1152–1157. 11257658

[3] Paez JG, Janne PA, Lee JC, Tracy S, Greulich H, et al (2004) EGFR mutations in lung cancer: correlation with clinical response to gefitinib therapy. Science 304: 1497–1500. 15118125 10.1126/science.1099314

[4] Lynch TJ, Bell DW, Sordella R, Gurubhagavatula S, Okimoto RA, et al (2004) Activating mutations in the epidermal growth factor receptor underlying responsiveness of non-small-cell lung cancer to gefitinib. N Engl J Med 350: 2129–2139. 15118073 10.1056/NEJMoa040938

[5] Blehm KN, Spiess PE, Bondaruk JE, Dujka ME, Villares GJ, et al (2006) Mutations within the kinase domain and truncations of the epidermal growth factor receptor are rare events in bladder cancer: implications for therapy. Clin Cancer Res 12: 4671–4677. 16899617 10.1158/1078-0432.CCR-06-0407

[6] Kassouf W, Black PC, Tuziak T, Bondaruk J, Lee S, et al (2008) Distinctive expression pattern of ErbB family receptors signifies an aggressive variant of bladder cancer. J Urol 179: 353–358. 18006009 10.1016/j.juro.2007.08.087 PMC2680144

[7] Kassouf W, Dinney CP, Brown G, McConkey DJ, Diehl AJ, et al (2005) Uncoupling between epidermal growth factor receptor and downstream signals defines resistance to the antiproliferative effect of Gefitinib in bladder cancer cells. Cancer Res 65: 10524–10535. 16288045 10.1158/0008-5472.CAN-05-1536

[8] Fajas L, Auboeuf D, Raspe E, Schoonjans K, Lefebvre AM, et al (1997) The organization, promoter analysis, and expression of the human PPARgamma gene. J Biol Chem 272: 18779–18789. 9228052 10.1074/jbc.272.30.18779

[9] Motomura W, Okumura T, Takahashi N, Obara T, Kohgo Y (2000) Activation of peroxisome proliferator-activated receptor gamma by troglitazone inhibits cell growth through the increase of p27KiP1 in human. Pancreatic carcinoma cells. Cancer Res 60: 5558–5564. 11034103

[10] Mansure JJ, Nassim R, Kassouf W (2009) Peroxisome proliferator-activated receptor gamma in bladder cancer: a promising therapeutic target. Cancer Biol Ther 8: 6–15. 19417560 10.4161/cbt.8.7.7853

[11] Guan YF, Zhang YH, Breyer RM, Davis L, Breyer MD (1999) Expression of peroxisome proliferator-activated receptor gamma (PPARgamma) in human transitional bladder cancer and its role in inducing cell death. Neoplasia 1: 330–339. 10935488 10.1038/sj.neo.7900050 PMC1508103

[12] Suzuki T, Nakagawa T, Endo H, Mitsudomi T, Masuda A, et al (2003) The sensitivity of lung cancer cell lines to the EGFR-selective tyrosine kinase inhibitor ZD1839 (‘Iressa’) is not related to the expression of EGFR or HER-2 or to K-ras gene status. Lung Cancer 42: 35–41. 14512185 10.1016/s0169-5002(03)00278-2

[13] Burgermeister E, Tencer L, Liscovitch M (2003) Peroxisome proliferator-activated receptor-gamma upregulates caveolin-1 and caveolin-2 expression in human carcinoma cells. Oncogene 22: 3888–3900. 12813462 10.1038/sj.onc.1206625

[14] Xin X, Yang S, Kowalski J, Gerritsen ME (1999) Peroxisome proliferator-activated receptor gamma ligands are potent inhibitors of angiogenesis in vitro and in vivo. J Biol Chem 274: 9116–9121. 10085162 10.1074/jbc.274.13.9116

[15] Bishop-Bailey D, Hla T (1999) Endothelial cell apoptosis induced by the peroxisome proliferator-activated receptor (PPAR) ligand 15-deoxy-Delta12, 14-prostaglandin J2. J Biol Chem 274: 17042–17048. 10358055 10.1074/jbc.274.24.17042

[16] Kassouf W, Chintharlapalli S, Abdelrahim M, Nelkin G, Safe S, et al (2006) Inhibition of bladder tumor growth by 1,1-bis(3′-indolyl)-1-(p-substitutedphenyl)methanes: a new class of peroxisome proliferator-activated receptor gamma agonists. Cancer Res 66: 412–418. 16397256 10.1158/0008-5472.CAN-05-2755

[17] Lee SY, Hur GY, Jung KH, Jung HC, Kim JH, et al (2006) PPAR-gamma agonist increase gefitinib's antitumor activity through PTEN expression. Lung Cancer 51: 297–301. 16386327 10.1016/j.lungcan.2005.10.010

[18] Zhou Y, Zheng S, Lin J, Zhang QJ, Chen A (2007) The interruption of the PDGF and EGF signaling pathways by curcumin stimulates gene expression of PPARgamma in rat activated hepatic stellate cell in vitro. Lab Invest 87: 488–498. 17372590 10.1038/labinvest.3700532

[19] Dinney CP, Fishbeck R, Singh RK, Eve B, Pathak S, et al (1995) Isolation and characterization of metastatic variants from human transitional cell carcinoma passaged by orthotopic implantation in athymic nude mice. J Urol 154: 1532–1538. 7658585

[20] Sabichi A, Keyhani A, Tanaka N, Delacerda J, Lee IL, et al (2006) Characterization of a panel of cell lines derived from urothelial neoplasms: genetic alterations, growth in vivo and the relationship of adenoviral mediated gene transfer to coxsackie adenovirus receptor expression. J Urol 175: 1133–1137. 16469639 10.1016/S0022-5347(05)00323-X

[21] Ono M, Kuwano M (2006) Molecular mechanisms of epidermal growth factor receptor (EGFR) activation and response to gefitinib and other EGFR-targeting drugs. Clin Cancer Res 12: 7242–7251. 17189395 10.1158/1078-0432.CCR-06-0646

[22] Morrison RF, Farmer SR (1999) Role of PPARgamma in regulating a cascade expression of cyclin-dependent kinase inhibitors, p18(INK4c) and p21(Waf1/Cip1), during adipogenesis. J Biol Chem 274: 17088–17097. 10358062 10.1074/jbc.274.24.17088

[23] Zhu Y, Qi C, Korenberg JR, Chen XN, Noya D, et al (1995) Structural organization of mouse peroxisome proliferator-activated receptor gamma (mPPAR gamma) gene: alternative promoter use and different splicing yield two mPPAR gamma isoforms. Proc Natl Acad Sci U S A 92: 7921–7925. 7644514 10.1073/pnas.92.17.7921 PMC41258

[24] Pettaway CA, Pisters LL, Dinney CP, Jularbal F, Swanson DA, et al (1995) Sentinel lymph node dissection for penile carcinoma: the M. D. Anderson Cancer Center experience. J Urol 154: 1999–2003. 7500444

[25] Emmans VC, Rodway HA, Hunt AN, Lillycrop KA (2004) Regulation of cellular processes by PPARgamma ligands in neuroblastoma cells is modulated by the level of retinoblastoma protein expression. Biochem Soc Trans 32: 840–842. 15494029 10.1042/BST0320840

[26] Hau P, Kunz-Schughart L, Bogdahn U, Baumgart U, Hirschmann B, et al (2007) Low-dose chemotherapy in combination with COX-2 inhibitors and PPAR-gamma agonists in recurrent high-grade gliomas - a phase II study. Oncology 73: 21–25. 18332649 10.1159/000120028

[27] Shrader M, Pino MS, Brown G, Black P, Adam L, et al (2007) Molecular correlates of gefitinib responsiveness in human bladder cancer cells. Mol Cancer Ther 6: 277–285. 17237287 10.1158/1535-7163.MCT-06-0513

[28] Lin J, Chen A (2008) Activation of peroxisome proliferator-activated receptor-gamma by curcumin blocks the signaling pathways for PDGF and EGF in hepatic stellate cells. Lab Invest 88: 529–540. 18332871 10.1038/labinvest.2008.20 PMC2673570

[29] Tang QQ, Lane MD (1999) Activation and centromeric localization of CCAAT/enhancer-binding proteins during the mitotic clonal expansion of adipocyte differentiation. Genes Dev 13: 2231–2241. 10485846 10.1101/gad.13.17.2231 PMC316997

[30] Lewis JD, Ferrara A, Peng T, Hedderson M, Bilker WB, et al (2011) Risk of bladder cancer among diabetic patients treated with pioglitazone: interim report of a longitudinal cohort study. Diabetes Care 34: 916–922. 21447663 10.2337/dc10-1068 PMC3064051

[31] Dormandy J, Bhattacharya M, van Troostenburg de Bruyn AR (2009) Safety and tolerability of pioglitazone in high-risk patients with type 2 diabetes: an overview of data from PROactive. Drug Saf 32: 187–202. 19338377 10.2165/00002018-200932030-00002

