# Correction: A Novel Mechanism of PPAR Gamma Induction via EGFR Signalling Constitutes Rational for Combination Therapy in Bladder Cancer

**DOI:** 10.1371/annotation/50295123-3bb7-4916-812c-fa4ea5f09130

**Published:** 2013-05-10

**Authors:** Jose Joao Mansure, Roland Nassim, Simone Chevalier, Konrad Szymanski, Joice Rocha, Saad Aldousari, Wassim Kassouf

After we have conducted this work, it has been reported that one the cell lines used in our study (KU-7) was cross-contaminated with HeLa-cells. Nevertheless, the authors are very confident with our findings since all the experimental results were conducted with different cell lines in parallel with those obtained using KU-7. For instance, the baseline of PPARg and EGFR and the dose-response to PPARg agonists and EGFR inhibitor shown in figure 1, is represented in a panel of 9 bladder cancer cell lines. The effect of the combined therapy on proliferation, on EGFR downstream signaling, on p21 expression, on the induction of PPARg and CEBPb expression in response to different concentrations of Gefitinib, (Figure 2, 3, 5, 6 and 9) respectively, were performed with the urothelial carcinoma cell line UM-UC13 as well.

With regards to the in vivo experiment, similar results were obtained with theUM-UC13 cell line where tumor weights were only significantly reduced in the combination therapy as compared to the control group (mean weight 248 mg vs 300mg, p=0.049, see attached figure). There was no significant reduction in tumor weights with either drug alone when compared to placebo.

Finally, one of our main results that shows that the efficacy of combination therapy can be improved in a schedule-specific manner was conducted with three different urothelial cell lines, UM-UC3, UM-UC6 and UM-UC13. Therefore, the report about the cross-contamination of KU-7 does not affect our findings. Our analysis is robust and dependent on several cell lines where KU7 is just one element of the overall experimental evidence. 

